# Meteorological Risks in Doha 2019 Athletics World Championships: Health Considerations From Organizers

**DOI:** 10.3389/fspor.2019.00058

**Published:** 2019-11-12

**Authors:** Stéphane Bermon, Paolo Emilio Adami

**Affiliations:** ^1^LAMHESS, Université Côte d'Azur, Nice, France; ^2^Health and Science Department, International Association of Athletics Federations, Monaco, Monaco; ^3^Department of Movement, Human and Health Sciences, University of Rome “Foro Italico”, Rome, Italy

**Keywords:** athletics, risk, mitigation, contingency, heat-related illnesses, meteorologic condition

## Abstract

The Doha 2019 IAAF World Championships represent a challenge for athletes, workforce and spectators who could compete, work or attend under likely extreme meteorological conditions. This short article summarizes the methodology used by the IAAF and the Local Organizing Committee doctors to analyze and reduce risks, while complying as much as possible with existing recommendations or policies. The main steps to be completed are identification and description of weather-related risks, description and whenever possible testing of all their possible mitigation measures during test events, revision of these risks once mitigation implemented, and finally drafting a contingency plan for remaining exceptional and impactful occurrences. Such risk management methodology could apply to other sports, ideally from the host city selection to the delivery of the competitive event.

## Introduction

On November 2014, the International Association of Athletics Federations (IAAF) Council voted and chose the city of Doha (Qatar) to host the IAAF World Championships in 2019. The Qatari officials supporting this bid had already failed 2 years earlier and the 2017 IAAF World Championships were finally attributed to London. Although the IAAF Council debates remain undisclosed, it is likely that the proposal of shifting World Championships to late September–early October instead of August on one hand, and a refurbished air-conditioned Khalifa stadium (May 2017) on the other hand, probably helped the Qatari officials to finally obtain the organization of the 2019 IAAF World Championships^1^. However, organizing a major sporting event in Qatar during the fall remains a challenge from a climatic perspective (Hosokawa et al., 2018b). Indeed, some Athletics endurance events, such as race walk and marathon competitions cannot be organized in an air-conditioned stadium, and therefore potentially expose athletes and staff to significant risks of heat-related illnesses.

## Sections on Policy Options and Implications

In order to guide member federations and Athletics competition organizers to provide suitable health care for athletes in and out of competition, the governing body of Athletics relies on Competition Medical Guidelines^2^. The guidelines' objectives are about providing appropriate and permanent medical care that will help athletes to reduce their risk of suffering from injuries and illnesses, and responding promptly to medical emergency situations. This document also provides guidance in designing the necessary services in order to offer excellent medical coverage to team officials, spectators and other members of the Athletics' family. Competition Medical Guidelines are not of mandatory application for the Local Organizing Committee (LOC) but are recommended to be followed. They serve, prior to and during the event, as a platform of discussion between the IAAF medical delegate and the chief medical officer from the LOC, should some country-specific adjustments need to be performed.

The IAAF Competition Medical Guidelines, chapter 3, paragraph 4.2 specifically deals with weather conditions. For the purpose of these guidelines, Wet Bulb Globe Temperature (WBGT) is used and calculated as follow:

WBGT = 0.7 WBT + 0.2 BGT + 0.1 DBT, where WBT, BGT, and DBT are wet bulb, black globe and dry bulb outdoor temperature, respectively (Yaglou and Minard, 1957).

A corresponding colored flag system can then be used to visually signal the thermal injury risk of current weather conditions to competitors and spectators (Table 1). For instance, a black flag means an extreme risk and corresponds to WBGT above 28°C. A red flag means a high risk and corresponds to WBGT between 23 and 28°C.

**Table 1:** The actual WBGT coding system described in the IAAF competition medical guidelines.

**Wet bulb globe temperature (WBGT) index flag coding system**	**WGBT**		**Recommendations**	
Black flag	Extreme	>28°C (>82°F)	Consider rescheduling or delaying the event until safer conditions prevail; if the event must take place, be on high alert
Red flag	High	23–28°C (73–82°F)	Everyone should be aware of injury potential; individuals at risk should not compete
Yellow flag	Moderate risk	18–23°C (65–73°F)	Risk increases as event progresses through the day
Green flag	Low	<18°C (<65°F)	Risk low but still exists on the basis of risk factors

Although this goes beyond the scope of this article, one must acknowledge that the use of WBGT in exercise and occupational physiology is debated (Budd, 2008; Brocherie and Millet, 2015). Indeed, there are some conceptual limitations in the WBGT as, for instance, it does not consider a possible restriction of sweating, the type of clothing and more importantly the level of endogenous caloric production associated with exercise.

Dealing with weather conditions, the IAAF guidelines also recommend that LOC medical and competition functional units should work together with the IAAF medical delegate to monitor weather conditions and that a specific contingency plan should be implemented to consider the scenario of extreme meteorological situations, that could force a delay or even a cancellation of the competition.

## Actionable Recommendations

As described below, there are four recommendations which should be actioned in the following order:

1. Assess the meteorological risks (definition, impact, and likelihood).
2. Set up a meteorological risks' mitigation strategy.
3. Reassess risks following implementation of the mitigation strategy.
4. Draft a contingency plan which addresses significant residual risks.

### Assessment of the Meteorological Risks

In Doha (Qatar), during fall, there are two types of adverse meteorological conditions to be considered. The first and main risk is represented by extremely hot and/or humid conditions. The second risk is the occurrence of dust storms.

Hot and humid weather conditions are represented in Table 2^3^. During late September and early October, mean WBGT is expected to be above 28°C (black flag) between 7 a.m. and 5 p.m. Should a heat wave (at least 2 consecutive days with unusually high level of air temperature) occur, this black flag period can be extended from 6 a.m. to midnight. During this period of the year, relative humidity is also expected to be from moderate to high, with the highest levels observed between 6 p.m. and 8 a.m.^4^ This relative humidity profile is negatively correlated with the level of solar exposure and radiation. From a thermoregulatory perspective, this is a dilemma since organizers must choose between the lesser of two evils; in this case high relative humidity.

**Table 2:**
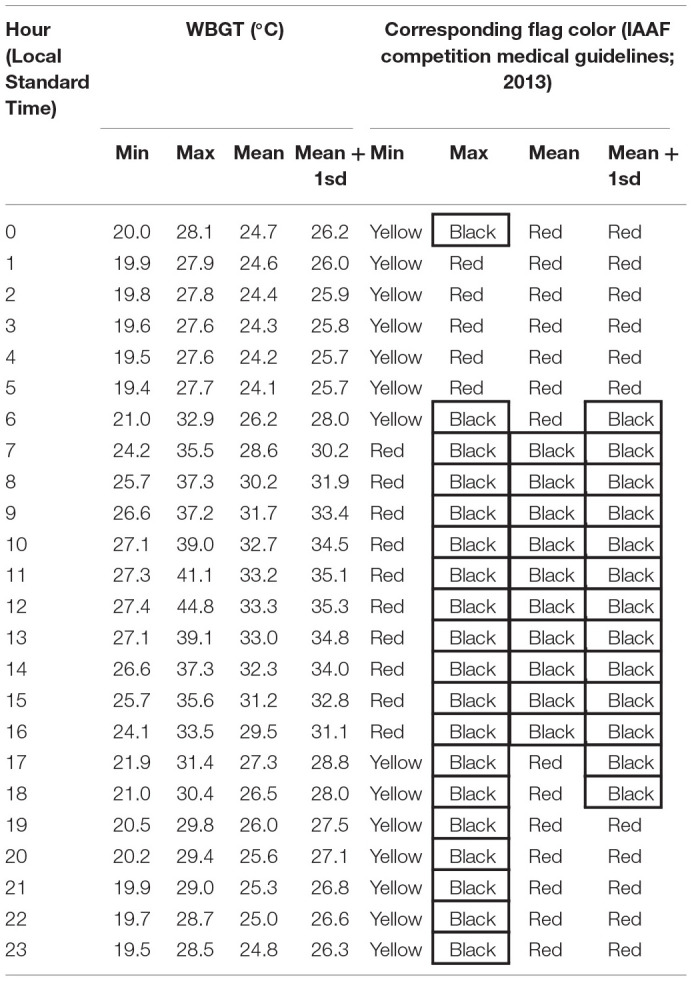
Risk assessment based on modern-era retrospective analysis for research and applications (MERRA-2) dataset recorded in Doha between 1980 and 2016.

*Hours are given in Local Standard Time and Min, Max, and Mean temperatures are expressed as average of values recorded between the 27th of September and the 7th of October (dates of the IAAF World Championships)*.

Dust storms are quite frequent in Qatar. The numerous deserts which engulf the Qatari peninsula represent infinite supplies of airborne solid particles, such as dust and sand. These spectacular phenomena associated with wind gusts, can carry large amounts of dust, with the leading edge being composed of a wall of thick dust as high as 1.6 km (Bartlett, 2004). In addition of their deleterious effects on visibility, engines, and electronic devices, exposure of human beings to desert dust include immediate increased respiratory symptoms and worsening of the lung function in individuals with asthma (Goudies, 2014). Acute *keratoconjunctivitis sicca* (“dry eyes”) can also occur in such circumstances (Goudies, 2014). In the Qatari area, these dust storms are called “*shamal*.” According to Bartlett (2004), “*shamal*” events mostly occur between March and September with a peak around May. In late September–early October, the monthly average occurance of “*shamal*” days is <2. Moreover, the most likely time of the day in terms of occurrence is 10 a.m. with mean duration of 4 h. However, the maximal duration can be up to 10 h which would possibly jeopardize race preparation and its execution. The wind associated with these events can be strong, but their average speed is 7.5 m/s.

The impact of a high WBGT on athlete is potentially very high. It goes from the benign consequences, such as exercise-associated muscle cramp and reduction in performance, to more serious medical conditions, such as heat exhaustion and exertional heat stroke (Epstein and Yanovich, 2019). Indeed, the most serious condition to occur is exertional heat stroke which can ultimately lead to death of athlete or staff if not properly and timely diagnosed and managed (Navarro et al., 2017; Hosokawa et al., 2018a). To a lesser level of gravity, heat stress is also associated with impaired athletic performance, especially in middle and long distance running and race-walking competitions (Guy et al., 2015). LOC volunteers or spectators, if exposed to heat for a long period of time (directing spectators or athletes, queuing), could suffer from non-exertional heat stroke.

In an Athletics competition setting, the impact of a dust storm is limited. Indeed, a healthy athlete could experience at most an asthma exacerbation, pulmonary symptoms, and conjunctivitis. Consequence of such a storm, which cannot be mitigated by using a protective mask, might be worst for unfit staff or workforce. This could prevent the individual from competing/working but is unlikely to represent a life-threatening condition. Conversely, potential impact on the competition logistic is very high. It could also seriously damage electronic devices that are used for timing, communication and broadcasting purposes as well as temporary installation, such as tent, banners, scaffoldings. The Khalifa stadium, which is not a fully covered stadium will be affected by a dust storm. Therefore, competitions held in this arena will have to be postponed for 12–24 h waiting for the end of the episode and the complete cleaning of the field of play. The Khalifa ventilation/air conditioning system is protected from this sand/dust and pathogen contamination by a complex system of progressive air filtering.

The likelihood of a high WBGT was calculated from the climatological data provided by the Qatari Civil Aviation Authority. In order to make the calculation of the cumulative distribution function, we have checked that temperatures recorded and provided by the Qatari Civil Aviation Authority, during 24 h in fall in Doha are normally distributed. Then, using the dataset reported in Table 2, the probability of a mean (calculated on 1 h) WBGT above 23°C WBGT is extremely high (>99.9%) between 7 a.m. and 5 p.m. A probability for a temperature above 28°C WBGT is high (64.6%) between 7 a.m. and 5 p.m.

#### Dust Storm

Dust storms are not frequent in late September–early October. Indeed, when the whole Qatari territory is considered, their occurrence is ~1.4 days per month (somewhere in the country) during that period of the year. Moreover, new meteorological models can predict these storms within a 48–72 h in advance (Bartlett, 2004). However, the 10 days duration of the Athletics World Championships increases this risk, which remains however low.

### Meteorological Risks Mitigation Strategy

A risk mitigation strategy, by definition, should take steps to reduce the risks components, i.e., their probability of occurrence and their impact.

One of the very first measures potentially to reduce the likelihood of high WBGT is to have most of the Athletics events organized in the Khalifa stadium, which is a unique stadium with an air conditioning system. Indeed, the ventilation system of this open stadium can be adjusted to WBGT as low as 20°C, while the outdoor WBGT is above 35°C. Unfortunately, race walking events and marathons cannot be held in stadium and cannot benefit from this revolutionary air conditioning system, which controls not only ambient temperature but also relative humidity. Therefore, shifting the dates of the World Championships from August to late September–early October has been the second mitigation measure proposed by the Qatari organizers to the IAAF. A third measure adopted by the IAAF was to propose to change the habitual competition timetable. As a result, all morning session were canceled and only evening sessions (heats and finals) were planned. Although the stadium has an air conditioning system, which will be set up between 23 and 25°C WBGT, the warm-up and training areas nearby (Aspire Zone) do not propose similar thermal conditions for all athletes, especially for distance runners and long throwers (discus, hammer throw, javelin). The most innovative measure (never used in any previous World Championships) was probably to move the start time of endurance events like marathons and race-walking competition very late at night (Table 3). Indeed, these events are usually planned early morning, between 6 and 9 a.m. during most of the IAAF World Championships. A quick look at Table 2 shows that this was not possible in this case, as most, if not all, of these events would have been initiated under black flag conditions, with a rising air temperature. An alternative solution would have been to start these events at 7 p.m., but the IAAF Health and Science Department advised its Competition Department and the LOC to take a safer approach and finally agreed on a start time after 11:30 p.m.

**Table 3:** Doha 2019 World Championships timetable (partial).

**Day 1–Friday, 27 Sep**				**Day 2–Saturday, 28 Sep**				**Day 3–Sunday, 29 Sep**				**Day 4–Monday, 30 Sep**			
16:30	Long Jump	M	Q A + B	16:15	Discus Throw	M	Q A	*19:40*	*50 km RW*	*W*	*MC*	16:30	Javelin Throw	W	Q A
16:35	100 m	M	Prelim	16:30	100 m	W	R1	*19:45*	*50 km RW*	*M*	*MC*	17:05	200 m	W	R1
16:40	Hammer Throw	W	Q A	17:05	800 m	M	R1	*19:50*	*Hammer Throw*	*W*	*MC*	18:00	Javelin Throw	W	Q B
17:10	800 m	W	R1	17:30	Pole Vault	M	Q A+B	20:05	200 m	M	R1	18:20	400 m	W	R1
17:30	Pole Vault	W	Q A + B	17:45	Discus Throw	M	Q B	20:40	Pole Vault	W	Final	*19:10*	*20 km RW*	*W*	*MC*
18:05	100 m	M	R1	18:05	400 m H	M	SF	*21:05*	*10,000 m*	*W*	*MC*	*19:15*	*Pole Vault*	*W*	*MC*
18:10	Hammer Throw	W	Q B	18:45	100 m	M	SF	21:20	100 m	W	SF	*19:20*	*4 × 400 m Relay*	*X*	*MC*
18:40	High Jump	W	Q A + B	*19:05*	*Marathon*	*W*	*MC*	21:45	Triple Jump	M	Final	*19:30*	*Triple Jump*	*M*	*MC*
19:00	3,000 m SC	W	R1	19:15	800 m	W	SF	21:55	800 m	M	SF	*19:35*	*100 m*	*W*	*MC*
19:25	Triple Jump	M	Q A + B	19:25	Hammer Throw	W	Final	*22:20*	*Long Jump*	*M*	*MC*	20:05	110 m H	M	R1
19:55	5,000 m	M	R1	20:00	4 × 400 m Relay	X	R1	22:35	4 × 400 m Relay	X	Final	20:30	High Jump	W	Final
20:30	400 m H	M	R1	20:40	Long Jump	M	Final	*22:40*	*100 m*	*M*	*MC*	20:50	200 m	M	SF
				21:10	10,000 m	W	Final	23:20	100 m	W	Final	21:20	5,000 m	M	Final
				22:15	100 m	M	Final					21:25	Discus Throw	M	Final
												21:50	3,000 m SC	W	Final
**Day 1–Friday, City, 27-28 Sep**				**Day 2–Saturday, City, 28-29 Sep**				**Day 3–Sunday, City, 29-30 Sep**				22:10	800 m	W	Final
*tbc*	*Opening*			23:30	50 km Race Walk	W	Final	23:30	20 km Race Walk	W	Final	22:40	400 m H	M	Final
23:59	Marathon	W	Final	23:30	50 km Race Walk	M	Final								
**Day 6–Wednesday, 2 Oct**				**Day 7–Thursday, 3 Oct**				**Day 8–Friday, 4 Oct**				**Day 9–Saturday, 5 Oct**			
16:35	100 m Dec	M		16:35	110 m H Dec	M		*19:45*	*Shot Put*	*W*	*MC*	16:30	Javelin Throw	M	Q A
16:45	Shot Put	W	Q A + B	16:40	Triple Jump	W	Q A + B	*19:50*	*400 m*	*W*	*MC*	17:15	100 m H	W	R1
17:05	100 m H Hep	W		17:30	Discus Throw Dec	M	A	20:10	1,500 m	M	SF	*17:25*	*400 m H*	*W*	*MC*
17:30	Long Jump Dec	M	A + B	*17:50*	*200 m*	*W*	*MC*	20:15	High Jump	M	Final	17:30	400 m	M	MC
17:35	1,500 m	W	R1	18:15	Long Jump Hep	W	A + B	20:40	4 × 100 m Relay	W	R1	17:50	Long Jump	W	Q A + B
18:00	Discus Throw	W	Q A	18:35	Discus Throw Dec	M	B	*20:50*	*Heptathlon*	*W*	*MC*	18:00	Javelin Throw	M	Q B
*18:10*	*Pole Vault*	*M*	*MC*	19:05	Pole Vault Dec	M	A	21:00	Discus Throw	W	Final	*19:05*	*Discus Throw*	*W*	*MC*
18:15	High Jump Hep	W	A + B	*19:15*	*Hammer Throw*	*M*	*MC*	21:05	4 × 100 m Relay	M	R1	*19:10*	*20 km RW*	*M*	*MC*
18:25	5,000 m	W	R1	19:20	Shot Put	M	Q A	21:30	400 m H	W	Final	19:55	4 × 400 m Relay	W	R1
18:50	Shot Put Dec	M	A + B	20:05	Pole Vault Dec	M	B	21:45	3,000 m SC	M	Final	20:05	Shot Put	M	Final
*19:15*	*800 m*	*M*	*MC*	20:10	Javelin Throw Hep	W	A + B	*21:55*	*Decathlon*	*M*	*MC*	20:25	4 × 400 m Relay	M	R1
19:25	Discus Throw	W	Q B	20:40	Shot Put	M	Q B	22:20	400 m	M	Final	20:35	Triple Jump	W	Final
20:05	110 m H	M	SF	*21:40*	*110 m H*	*M*	*MC*	*22:25*	*3,000 m SC*	*M*	*MC*	20:55	1,500 m	W	Final
*20:25*	*Javelin Throw*	*W*	*MC*	22:00	1,500 m	M	R1	*22:30*	*High Jump*	*M*	*MC*	21:25	5,000 m	W	Final
20:30	Shot Put Hep	W	A + B	22:05	Javelin Throw Dec	M	A					*21:55*	*Shot Put*	*M*	*MC*
20:35	400 m	M	SF	22:35	Shot Put	W	Final					22:05	4 × 100 m Relay	W	Final
20:40	High Jump Dec	M	A + B	23:00	1,500 m	W	SF					22:15	4 × 100 m Relay	M	Final
21:05	400 m H	W	SF	23:10	Javelin Throw Dec	M	B					*22:20*	*1,500 m*	*W*	*MC*
21:30	200 m	M	MC	23:50	400 m	W	Final								
21:40	Hammer Throw	M	Final	00:05	800 m Hep	W	Final								
21:50	200 m Hep	W		00:25	1,500 m Dec	M	Final								
22:35	200 m	W	Final					**Day 8–Friday, City, 4-5 Oct**				**Day 9–Saturday, City, 5-6 Oct**			
22:55	110 m H	M	Final					23:30	20 km Race Walk	M	Final	23:59	Marathon	M	Final
23:15	400 m Dec	M													

*Italic values represent Medal Ceremony (MC). Gray shades highlight the timing of endurance events held at the Corniche: Race Walks and Marathons*.

An important measure taken to reduce the impact of high WBGT is to set up an education and communication campaign in the direction of athletes, their supporting staff as well as volunteers and workforce. This communication plan is achieved through various means, such as conferences for doctors, coaches and athletes, IAAF's circular letters to its member federations, electronic and printed leaflets^5^ distributed by the IAAF (including through its website) and the LOC. The main topics addressed in this campaign are:

- The anticipated climatic condition in Doha during the World Championships,
- Basic notion of thermoregulation in exercising individuals,
- How does heat impair health and performance,
- The importance of hydration (qualitative and quantitative aspects) and how to identify dehydration,
- How and when to heat-acclimatize,
- Description and benefits of main pre- and per-cooling methods.
- Additional heat-illness risk factors, such as consumption of stimulants, diuretics, and non-steroidal anti-inflammatory drugs

Another set of mitigation actions to reduce the impact of high WBGT, consisted of secondary prevention measures. These are mainly for health professionals, involved in the World Championships medical plan (national teams and LOC). Some of the team doctors, physiotherapists, nurses and athletic trainers were offered to attend continuing educational meetings and practical workshops organized by the IAAF Health and Science Department and the International Institute for Race Medicine^6^. This educational program contains pedagogical material that specifically deals with heat-related medical condition diagnoses and management. Similarly, all LOC health professionals underwent similar education session under the supervision of the LOC chief medical officer. Finally, extensive preparation work was done to build and organize high end medical stations on the site of the competitions. Exertional heat stroke represents the most serious medical condition that the medical staff could have to early diagnose and treat on the competition site, as an immediate transfer to an hospital setting is not recommended (Flouris et al., 2015). Indeed, heat stroke patients (core temperature above 40.5°C at the time of the collapse) should be immediately immersed in ice bath (water temperature below 10°C) while monitoring the decrease of their core temperature through a rectal thermistor (Flouris et al., 2015) This the reason why the Corniche main medical station, where marathons and race-walking events are planned were voluntarily oversized and overequipped to host and treat on site (including serious heat-related illnesses) a maximum of twenty four athletes in a 1-h period. The maximal number of athletes competing at the same time is 80 in the 50 km race walking men and women. This ratio between the number of medical and the number of athletes on the race course has never existed in any previous IAAF World Championships. The marathon races are run on seven laps of 6,027 m. Twenty and 50 km race walks use the appropriate number of 1 and 2 km laps, respectively. As all these laps are the two sideways of a main (Corniche) coastal road, surveillance, alert, and intervention of the medical staff, and transfer to secondary of main medical settings is very fast and facilitated.

Deciding to hold the World Championships in late September–early October, as well as holding the competitive events in the evening, when these phenomena are at their minimum occurrence rate, is the only option which was taken by both the LOC and the IAAF to reduce the likelihood of dust storms.

Regular contacts between the LOC and the Qatari meteorological authorities are fundamental. Although the impact of a dust storm on athlete is limited, the consequences on other sectors of the organization for which visibility and sensible electronic material are important (broadcasting, timing, marketing, security) can be quite significant but are beyond the scope of this article.

### Reassessment of Risks Following Implementation of the Mitigation Strategy

As shown in Table 3, start time for all the endurance events organized out of the air-conditioned Khalifa stadium has been set between 11:30 p.m. and midnight. This was decided following a new calculation of the likelihood of high WBGTs with these new schedules. Recalculating the cumulative distribution function (Table 2), the likelihood of facing a red flag at midnight (approximate start time) during marathons or race walk events of the World Championships is very high at 87%. However, a start can be given under a red flag condition. Similarly, the likelihood of facing a black flag at midnight during marathons or race walk events is ~2.4%. Giving a start under black flag condition can be problematic as it is not in accordance with the IAAF Competition Medical Guidelines, expose athletes to heat-related illnesses, and decreased performance. One can see here that moving the endurance race start time from 7 a.m. to midnight is a very efficient mitigation measure that reduces the likelihood of a cancellation or a rescheduling associated with a black flag. Indeed, this probability moves from 64.6% down to 2.4%, whereas it modestly affects the risk linked to a red flag (from almost 100% to 87%).

### Draft a Contingency Plan Which Addresses Significant Residual Risks

In the rare occurrence of a WBGT above or equal to 28°C at the start of marathons or race-walking events, a contingency plan could be implemented. Here below are very brief descriptions of practical measures that the LOC and the IAAF Competition and Medical Directors could take. For all below events, a major measure would be to stop all athletes still competing on the race course after 6:15 a.m., since a significant rise in WBGT occurs after this time (Table 2). This also means that if postponing the start of the competition is considered, the expected chronometric performance of an average elite athlete under extreme heat conditions, must be considered in the calculation of the new start time. Finally, delaying the start of any of the race-walking and marathon events by more than 2 h, whereas convenient from a pure meteorological point of view is quite irrelevant for the athletes as it may seriously interfere with their preparation, warm-up and nutrition plans.

#### Women Marathon

Start time (23:59) could be delayed until having more favorable conditions and up to 2:00 a.m. If the solution above cannot be implemented it is recommended to reschedule the women marathon race the same day and time as the men marathon race.

#### Men Marathon

Start time (23:59) could be delayed until having more favorable conditions and up to 2 a.m.

#### Women and Men 50 km Race Walks

Start time (23:30) could be delayed until having better thermal conditions and up to 1:15 a.m. (women) and 1:30 (men). The women 50 km Race Walk is likely to be the most difficult to design a contingency plan for, as it is the longest Athletics event in duration. If a delay is impossible, alternate solutions are to organize the race on day 3 after the women 20 km Race Walk (unlikely in the event of a lasting heat wave), or on day 8 after the men 20 km Race Walk, or ultimately to shorten the distance of the event. In any case the start time should not be after 1:30 a.m., if the distance of 50 km is maintained.

#### Women 20 km Race Walk

Start time (23:30) must be delayed until having more favorable conditions and up to 1:30 a.m.

#### Men 20 km Race Walk

Start time (23:30) should be delayed until having more favorable conditions and up to 1:30 a.m.

The occurrence of a very unlikely dust storm right before out of stadium endurance events, should be dealt on the same way as the extreme heat risk (see above).

Setting up a Crisis Unit and describing its activation process is an important component of the contingency plan. The Crisis Unit consists of the IAAF and LOC Chief Executive Officer, the IAAF and LOC Medical Delegate, the IAAF Competition, Communication and Broadcasting Departments Directors. Although the IAAF Competition Rules 113^7^ gives the IAAF Medical Delegate the power to order one or several athletes to withdraw before, or to immediately retire from an event during, competition, extreme meteorological conditions will trigger the activation of the Crisis Unit. Ideally, this activation should be anticipated as both heat waves and dust storms can be predicted or suspected at least 48 h before their occurrence. Therefore, it is important that a regular prediction and monitoring of these meteorological conditions is organized by the LOC.

## Conclusions

**Figure 1:**
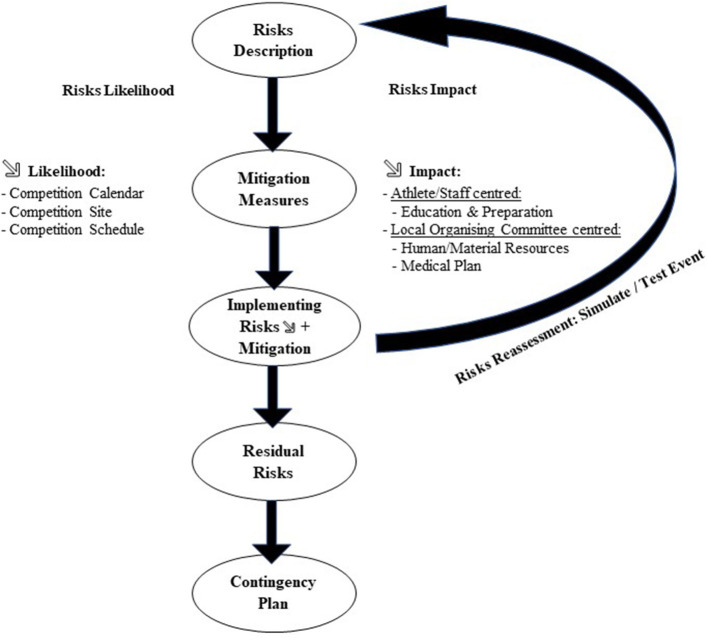
Schematic view of meteorological risks assessment and management prior to Doha 2019 IAAF World Championships.

Globalization and climate changes accelerated during the last 15 years, increasing the likelihood of sports governing bodies to organize major competitive events in locations where extreme weather conditions may be encountered. Sports policies and regulations may insufficiently or not at all address these extreme meteorological conditions and their potential deleterious consequences on athletes, workforce and spectators' health and safety. Therefore, it is important, while updating or drafting such health policies, to describe and assess all possible weather-related risks (Figure 1). This assessment should carefully consider the risks and impact on the competition. Then, specific measures and decisions that could reduce each risk and impacts should be listed and prioritized. The next step should consist on a revision of the climatic risks, after implementing or testing all mitigation measures. This reassessment should be theoretical and if possible, practically done through a test event at a smaller scale. For the risks which remain significant, a contingency plan and a crisis decision-making process should be prepared (Figure 1).

## Author Contributions

SB and PA drafted and reviewed the manuscript, tables, and figure.

### Conflict of Interest

The authors declare that the research was conducted in the absence of any commercial or financial relationships that could be construed as a potential conflict of interest.

## Footnotes

^1^https://www.iaaf.org/news/press-release/iaaf-evaluation-commission-candidates-2019-ia

^2^IAAF Competition Medical Guidelines: Available online at: https://www.iaaf.org/about-iaaf/documents/health-science

^3^https://gmao.gsfc.nasa.gov/reanalysis/MERRA-2/

^4^https://weatherspark.com/m/105083/10/Average-Weather-in-October-in-Doha-Qatar#Sections-Humidity

^5^Beat the Heat Leaflet: Available online at: https://www.iaaf.org/about-iaaf/documents/health-science

^6^International Institute for Race Medicine: Available online at: https://www.racemedicine.org/

^7^IAAF Competition Rules: Available online at: https://www.iaaf.org/about-iaaf/documents/rules-regulations
